# Animal Study on Primary Dysmenorrhoea Treatment at Different Administration Times

**DOI:** 10.1155/2015/367379

**Published:** 2015-02-02

**Authors:** Bao-Chan Pu, Ling Fang, Li-Na Gao, Rui Liu, Ai-zhu Li

**Affiliations:** ^1^Tianjin State Key Laboratory of Modern Chinese Medicine, School of Traditional Chinese Materia Medica, Tianjin University of Traditional Chinese Medicine, Tianjin 300193, China; ^2^Research Center of Traditional Chinese Medicine, Tianjin University of Traditional Chinese Medicine, Tianjin 300193, China

## Abstract

The new methods of different administration times for the treatment of primary dysmenorrhea are more widely used clinically; however, no obvious mechanism has been reported. Therefore, an animal model which is closer to clinical evaluation is indispensable. A novel animal experiment with different administration times, based on the mice oestrous cycle, for primary dysmenorrhoea treatment was explored in this study. Mice were randomly divided into two parts (one-cycle and three-cycle part) and each part includes five groups (12 mice per group), namely, Jingqian Zhitong Fang (JQF) 6-day group, JQF last 3-day group, Yuanhu Zhitong tablet group, model control group, and normal control group. According to the one-way ANOVAs, results (writhing reaction, and PGF_2*α*_, PGE_2_, NO, and calcium ions analysis by ELISA) of the JQF cycle group were in accordance with those of JQF last 3-day group. Similarly, results of three-cycle continuous administration were consistent with those of one-cycle treatment. In conclusion, the consistency of the experimental results illustrated that the novel animal model based on mice oestrous cycle with different administration times is more reasonable and feasible and can be used to explore in-depth mechanism of drugs for the treatment of primary dysmenorrhoea in future.

## 1. Introduction

Dysmenorrhoea, which is caused by female endocrine disorders, is one of the unresolved problems in medical science. Approximately 80% of women globally had dysmenorrhoea in different degrees according to a report from the British medical authority [1]. This condition is classified into two categories: primary and secondary dysmenorrhoea. The incidence of primary dysmenorrhoea is higher. In an epidemiological study, 53.2% of women in China have dysmenorrhoea during their menstrual period, and the incidence rate is about 43% to 70% overseas [2]. Primary dysmenorrhoea can be mostly experienced by young women, which occurs without an identifiable pelvic pathology. Such condition is mainly characterised by cramping pain in the lower abdomen immediately before or during menstruation, which does affect the quality of their life and work [3].

Nonsteroidal anti-inflammatory drugs (NSAIDs) are the most commonly used drugs for primary dysmenorrhoea treatment [4], particularly for cases in which subjective symptoms are moderate. However, NSAIDs have side effects, such as damage of liver and kidney after discontinuing medication [5].

Chinese medical treatment of primary dysmenorrhoea has a long history. Based on the rich clinical experience and syndrome differentiation theory, primary dysmenorrhoea was recently divided into five types, including the qi stagnation and blood stasis type, cold-damp stagnation type, qi and blood deficiency type, and liver and kidney deficiency type [6]. Traditional Chinese medicine (TCM) treats primary dysmenorrhoea by fundamentally regulating the female physique and body balance of Yin and Yang, and TCM mostly has no obvious side effects. Therefore, such treatment has elicited considerable attention worldwide.

Jingqian Zhitong Fang (JQF) prescription is a typical and relatively simple TCM formula. Such prescription originally came from Fo Shou San and has been proven effective for treating primary dysmenorrhoea. Radix Angelicae Sinensis (*Angelica sinensis* Diels) is the main drug in JQF and the main constituent that affects blood [7]. It could enrich the blood and alleviate menstrual pain [8]. Rhizoma Chuan Xiong (*Ligusticum chuanxiong* Hort.) can treat menstruation, amenorrhea, dysmenorrhoea, and headache [9]. Herba Leonuri Motherwort (*Leonurus japonicus* Houtt.) can also regulate menstruation [10]. Radix Paeoniae Rubra (*Paeonia lactiflora* Pall.) is used in clinical treatment of heat and cold blood, as well as for healing bruises and relieving pain [11]. We developed an ultra-performance liquid chromatography-quadrupole time-of-flight mass spectrometry (UPLC/Q-TOF-MS) method to construct the chromatographic fingerprint and to identify the constituents of JQF. Based on experimental pharmacological studies [12–14], JQF has a wide variety of actions, including antinociceptive, anti-inflammatory spasmolysis and vasorelaxation, all of which may contribute to its therapeutic action.

Some restricted problems such as slow onset, long treatment cycle (the period of TCM treatment is three months per course), and poor taste of drugs hinder the modernization and internationalization of TCM [15, 16]. Such problems need to be resolved. Recently, through continuous exploration, short-term medication and effective treatment method at different administration times for primary dysmenorrhoea are widely used clinically [17–21]. The administration time is 3 days (d) to 7 d before menstruation instead of the entire physiological cycle during medication. This treatment was only reported in clinical trials and never occurred in animal experiments. An animal experiment for primary dysmenorrhoea could facilitate not only observational changes of induced pain factor content but also an in-depth study of the pathway, receptor cells, and uterine tissues. After many years of discussion and research, animal experiments for primary dysmenorrhoea have already found a certain research foundation [22–24]. Animal experiments are important means for investigating the mechanisms of action underlying primary dysmenorrhoea.

Recently, animal studies have been conducted to explore the basis and mechanism of primary dysmenorrhoea treatment and developed a general animal model of this condition. Therefore, the current experiment selected the point of different administration times to treat primary dysmenorrhoea on the basis of the formal clinical studies [25–27]. The modelling time was selected in line with oestrous cycle by observing the mice vaginal smears and provided medicine in groups combined with clinical treatment. The scientific connotation of primary dysmenorrhoea treatment can then be explored at different administration times by animal experimental study. This treatment provides basic ideas for further research and also facilitates new information and methods for animal studies of primary dysmenorrhoea, which have explored the mechanism of action and dosage of drugs in recent years. Such treatment also provides further experimental data for TCM treatment of primary dysmenorrhoea and promotes modernisation and globalisation of TCM application.

In this study, we aimed to explore and establish an animal model by evaluating primary dysmenorrhoea treatment at different administration times, in which the primary dysmenorrhoea model was based on the proestrus stage of mice. Furthermore, the present study was a preliminary study on the mechanism of the treatment and optimized the animal primary dysmenorrhoea model.

## 2. Material and Methods

### 2.1. Experimental Animal

A total of 140 female Kunming mice (weighing 18 g to 22 g) without mating were purchased from the Beijing Hua Fukang Experimental Animal Technology Co., Ltd. (approval number: 2013-0004). The animal care complied with the United States National Institutes of Health Advocacy. The mice were housed in individual cages with food and water ad libitum in a 12 h : 12 h light/dark cycle with indoor temperature of 25°C and 45% humidity.

### 2.2. Experimental Equipment and Reagents

For intervention, oestradiol benzoate for injection (Ningbo Sansheng Pharmaceutical Co., Ltd., batch number: 120904), oxytocin for injection (Shanghai Hefeng Factory, batch number: 130115), and Yuanhu Zhitong tablets (Tianjin Tongrentang Group Limited by Share Ltd., batch number: c91017) were used.

For outcome, prostaglandin E_2_ assay kit, prostaglandin F_2*α*_ assay kit, nitric oxide assay kit (nitrate reductase method), and calcium colorimetric assay kit (Nanjing Jiancheng Bioengineering Institute); YXJ-type high speed centrifuge (Changzhou Jiancheng Teaching Instrument Factory); OLYMPUS U-CMAD3 optical microscope; Japanese OLYMPUS, OLYMPUS C5060-ADU optical camera; Japanese OLYMPUS; gas bath thermostats oscillator (Jintan Ronghua Instrument Manufacturing Co., Ltd.); and Infinite 200 PRO automatic enzyme mark instrument (Tecan Group, Ltd.) were applied.

### 2.3. Determination of Oestrous Cycle and Experimental Groups

All 140 mice were fasted for 2 d and were randomly divided into groups before starting the experiments. At 8:00 AM, the mice vaginal mucus were collected on a glass slide fixed with methanol and stained with HE to determine the oestrous cycle time and to divide the same oestrous cycle mice in a group [28]. As a result of individual differences of the oestrous cycle of mice, 128 mice were confirmed with an oestrous cycle of 6 d, among which 120 mice were selected for experiment.

**Figure 1:**
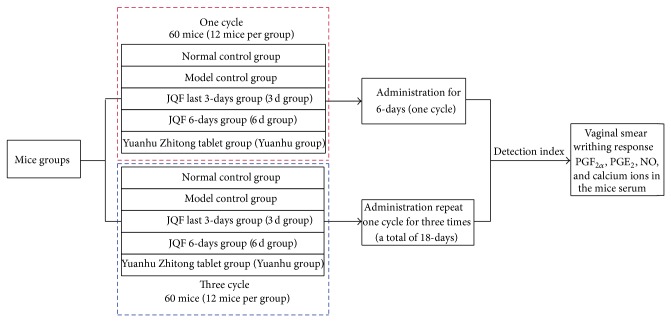
The experimental scheme. The entire experiments were departed into two parts to investigate the efficiency of treatment for one and three cycles. In each treatment cycle, mice were randomized into five groups, namely, JQF cycle group (6 d group), JQF last 3 d group (3 d group), Yuanhu Zhitong tablet group (Yuanhu group), model control group, and normal control group.

The entire experiments were departed into two parts to investigate the efficiency of treatment for one and three cycles. In each treatment cycle, mice were randomized into five groups, namely, JQF cycle group (6 d group), JQF last 3 d group (3 d group), Yuanhu Zhitong tablet group (Yuanhu group), model control group, and normal control group (Figure 1).

### 2.4. Primary Dysmenorrhoea Mice Model and Administration

The primary dysmenorrhoea mice model is improved from reference [29] of building the dysmenorrhoea model and assessment methods. Firstly, the model should determine the proestrus stage of the mice via vaginal smear test, and then dysmenorrhoea mice were modelled. In detail, oestradiol benzoate injection was subcutaneous injected to mice except those in the normal control group to establish the animal model of primary dysmenorrhoea. For the JQF last 3 d group (in one cycle, mice were given saline for the first 3 days, and JQF was replaced for the remaining last 3 days) and 6 d group, mice were intragastrically given JQF (0.218 g/kg). As a positive control, Yuanhu Zhitong tablet group (0.1 g/kg) were intragastrically administered (0.02 mL/g). Simultaneously, saline at the same dose was given to mice in the normal control group (Figure 1 and Table 1).

### 2.5. Writhing Response and Preparation for Determination

The drug effect on the behavioural pattern of mice was investigated. Writhing response complied with the Schmauss and Yaksh's [30] standard. The mice were fasted with water access for 12 h to 16 h prior to initiation of the study. On the sixth day, after the injection of oestradiol benzoate moulding for 40 min, the mice were administered oxytocin 2 U/mice (0.01 mL/g) by intraperitoneal injection. After 5 min, the writhing reaction times were recorded for 30 min. Blood was then extracted from the retroorbit sinus. Plasma specimens were separated by centrifugation at 3500 r·min^−1^ for 15 min at −4°C and then stored at −20°C until analysis. The specific method for sample detection strictly complied with the kit operation. The experiment complied with the Guide for the Care and Use of Laboratory Animals (National Research Council of the USA, 1996) and related ethical regulations of the China Academy of Chinese Medical Sciences.

### 2.6. Statistical Management

SPSS 13.0 software was used for statistical analysis. One-way ANOVA was used to analyse the difference between the two groups, and data were expressed as mean ± standard error of mean. A *P* value less than 0.05 or 0.01 was considered as significance.

## 3. Results

### 3.1. Stage of Oestrous Cycle Typical Vaginal Smear

**Figure 2:**
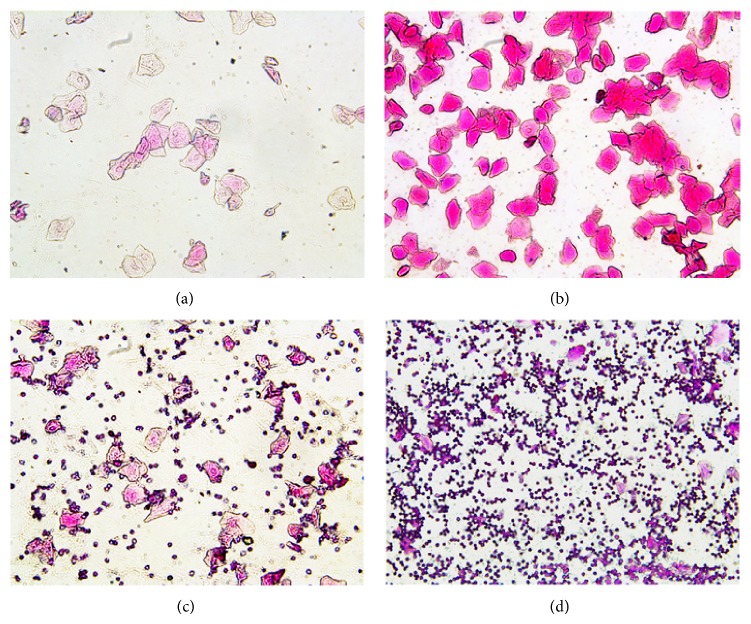
Mice typical vaginal smear of different stages of the oestrous cycle (200x). (a) Proestrus smear; (b) estrus smear; (c) metestrus smear; (d) diestrus smear.

The characteristic of proestrus smear was almost nucleated epithelial and occasionally had few keratinocytes (Figure 2(a)). The smear was almost entirely covered with nucleus-free keratinocytes in the estrus (Figure 2(b)). White blood cells, keratinocytes, and nucleated epithelial cells simultaneously existed in the metestrus (Figure 2(c)). The diestrus mainly had white blood cells (Figure 2(d)).

### 3.2. Effects of Different Administration Times on Oxytocin-Induced Dysmenorrhoea Mice Model

**Figure 3:**
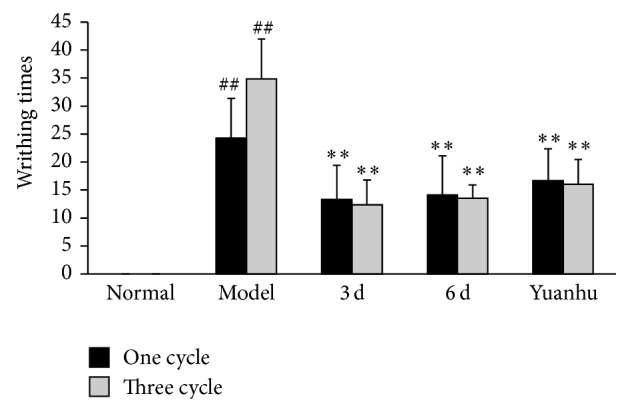
Effects of different administration times on oxytocin-induced dysmenorrhoea mice model. Values are means ± SD (*n* = 12) and significant difference compared with control group; ^##^ *P* < 0.01, model control group versus normal control group; ^**^ *P* < 0.01, 6 d and 3 d groups; Yuanhu group versus model control group.

Compared with the normal control group, the writhing times of mice in the model control group were significantly increased (*P* < 0.01) (Figure 3). Compared with the model control group, the writhing times of the mice were significantly decreased in the one-cycle parts (6 d and the last 3 d group, *P* < 0.01). Results of the three-cycle continuous moulding administration experiment were similar to those of the one-cycle treatment, but without significance between one- and three-cycle parts. Compared with the model control group, moreover, Yuanhu group decreased the writhing times of the mice (*P* < 0.05 or *P* < 0.01), not only in the one-cycle part but also in the three-cycle part.

### 3.3. Effects of PGF_2*α*_ and PGE_2_ at Different Administration Times on Dysmenorrhoea Mice

**Figure 4:**
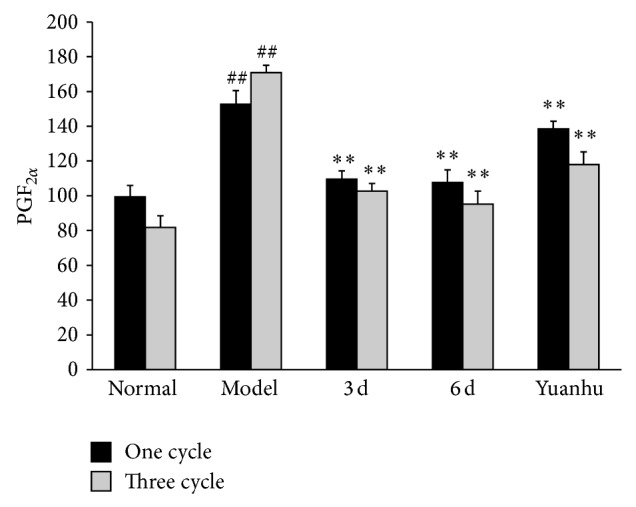
Effects of PGF_2*α*_ at different administration times on dysmenorrhoea mice. Values are means ± SD (*n* = 6) and significant difference compared with control group; ^##^ *P* < 0.01, model control group versus normal control group; ^**^ *P* < 0.01, 6 d and 3 d groups; Yuanhu group versus model control group.

**Figure 5:**
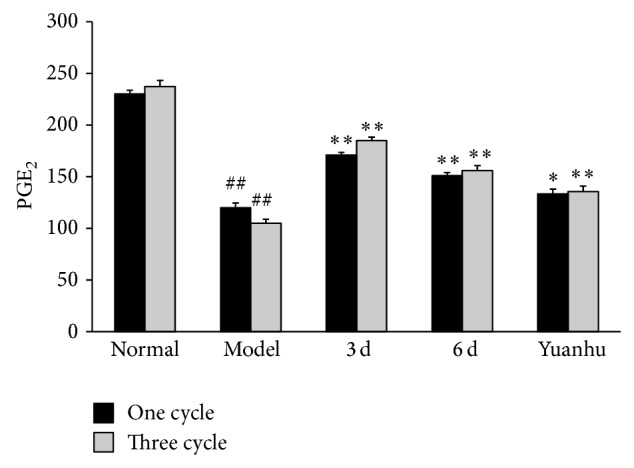
Effects of PGE_2_ at different administration times on dysmenorrhoea mice. Values are means ± SD (*n* = 6) and significant difference compared with control group; ^##^ *P* < 0.01, model control group versus normal control group; ^**^ *P* < 0.01, 6 d and 3 d groups versus model control group; Yuanhu group versus model control group in one cycle ^*^ *P* < 0.05 and three cycles ^**^ *P* < 0.01.

**Figure 6:**
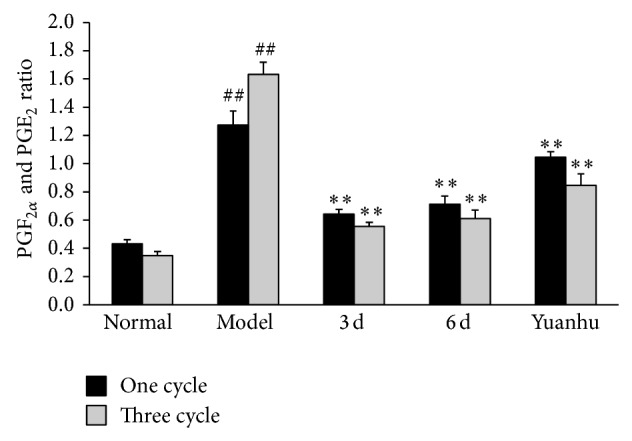
Effects of PGF_2*α*_ and PGE_2_ ratio at different administration times on dysmenorrhoea mice. Values are means ± SD (*n* = 6) and significant difference compared with control group; ^##^ *P* < 0.01, model control group versus normal control group; ^**^ *P* < 0.01, 6 d and 3 d groups versus model control group; Yuanhu Zhitong tablet group versus model control group only in three cycles ^**^ *P* < 0.01.

Compared with the normal control group, PGF_2*α*_ of the model control group was significantly increased, and PGE_2_ was significantly decreased, whereas the serum of PGF_2*α*_ and PGE_2_ ratio was significantly increased (*P* < 0.01). Compared with the model control group, PGF_2*α*_ was significantly decreased, and PGE_2_ was significantly increased; the serum of PGF_2*α*_ and PGE_2_ ratio tended to be normal in the 6 d group and the last 3 d group (*P* < 0.01). The results of the three-cycle continuous moulding administration experiment were similar to those of the one-cycle treatment. Compared with the model control group, the one-cycle experiment of the Yuanhu group decreased the PGF_2*α*_, whereas PGE_2_ increased (*P* < 0.05). However, no obvious difference was found in the serum of PGF_2*α*_ and PGE_2_ ratio. After three cycles of continuous administered treatment, the effects of all indexes were significantly modulated (*P* < 0.01) (Figures 4, 5, and 6).

### 3.4. Effects of NO and Calcium Ions at Different Administration Times on Dysmenorrhoea Mice

**Figure 7:**
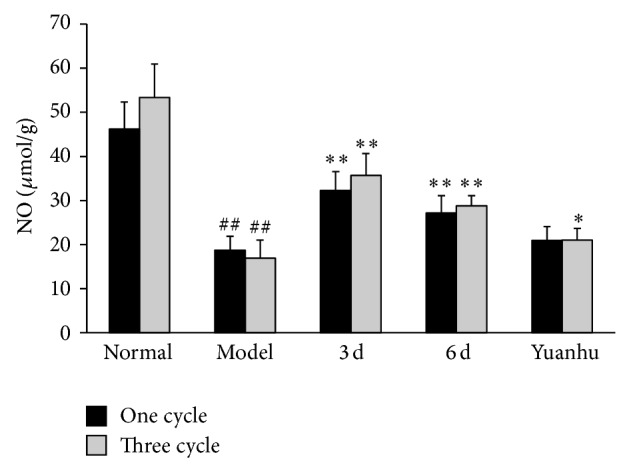
Effects of NO at different administration times on dysmenorrhoea mice. Values are means ± SD (*n* = 6) and significant difference compared with control group; ^##^ *P* < 0.01, model control group versus normal control group; ^**^ *P* < 0.01, 6 d and 3 d groups versus model control group; Yuanhu group versus model control group only in three cycles ^*^ *P* < 0.05.

**Figure 8:**
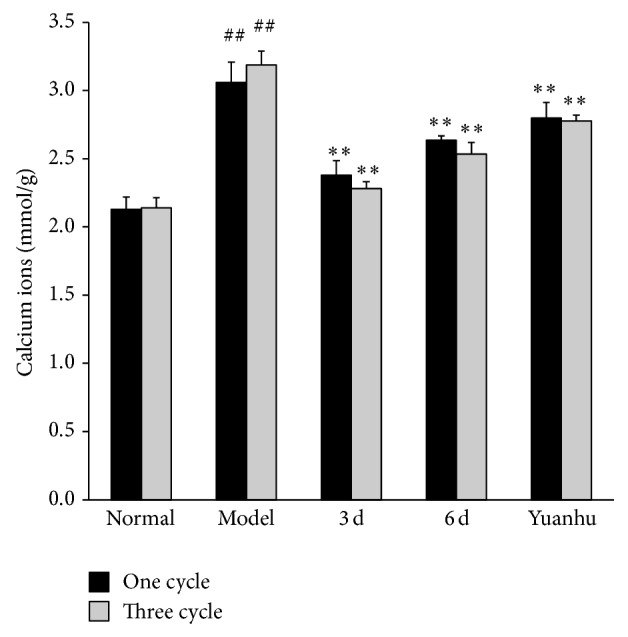
Effects of calcium ions at different administration times on dysmenorrhoea mice. Values are means ± SD (*n* = 6) and significant difference compared with control group; ^##^ *P* < 0.01, model control group versus normal control group; ^**^ *P* < 0.01, 6 d and 3 d groups; Yuanhu group versus model control group.

Compared with the normal control group, NO contents in the serum of the model control group were significantly decreased, whereas calcium ions were significantly increased (*P* < 0.01). Compared with the model control group, NO contents in the serum were significantly increased, whereas calcium ions were significantly decreased in the 6 d and the last 3 d group (*P* < 0.01). The results of the three-cycle continuous moulding administration experiment were similar to those of the one-cycle treatment. Compared with the model control group, the Yuanhu group significantly decreased the calcium ions in both one-cycle and three-cycle experiments (*P* < 0.01). However, no obvious difference was found in the NO contents of the serum in the one-cycle experiment. The NO contents were significantly increased after three cycles of continuous administered treatment (*P* < 0.05) (Figures 7 and 8).

## 4. Discussion

### 4.1. Screening of Experimental Animal and Basis of Another Administration Time

In this study, experimental animal selection was defined based on our previous study on vaginal smear oestrous cycle of female rats and mice. The oestrous cycle of mice was generally from 5 d to 6 d, whereas that of rats was from 4 d to 5 d [31]. Owing to the fact that each stage of the oestrous cycle of rats was shorter and almost the same, the drug cannot completely play its role, and the results may be affected. On the basis of the time of primary dysmenorrhoea in animal model, the mice need only 6 d, and the rat usually takes about 10 d [32], which is not conducive to perform three cycles of continuous administration experiment. Finally, the female mice were selected as the experimental animals.

The female physiology cycle is divided into four phases [33]: menstrual period (1 d to 4 d of the menstrual cycles); estrin phase (5 d to 14 d of the menstrual cycles, follicular maturation stage); progestational stage (15 d to 24 d of the menstrual cycles, corpus luteum maturation stage); and premenstrual period (25 d to 28 d of the menstrual cycles, degeneration of the corpus luteum period). In this study, on the basis of this theory and the result of JQF in previous clinical studies, the medication in luteal regression period of patients was effective, and the diestrus of mice was at the last 3 d of the cycle through vaginal smear. Therefore, the last 3 d in the cycle group (the last 3 d group) that equals administration in the corpus luteum period [34] was finally defined as another time of administration. To explore the different administration times of primary dysmenorrhoea, the results of the experiment cycle group (6 d group) were compared with those of the animal experiment.

### 4.2. Relationship between Related Pain Factors and Primary Dysmenorrhoea

This research determined four kinds of wide pain factors from the existing primary dysmenorrhoea animal experiment references to explore the scientific connotation of different administration times for primary dysmenorrhoea treatment from the mechanism perspective. Prostaglandin was the most relevant pain factor to primary dysmenorrhoea [35]. PGF_2*α*_ combined with PGF_2*α*_ receptor on the spiral arterioles increased uterine contractility, resulting in ischemia pain [36]; PGE_2_ could inhibit uterine contraction and induce uterine relaxation. The content of PGF_2*α*_/PGE_2_ in endometrium of dysmenorrhoea patients is higher than that of healthy women [37]. NO influenced the primary dysmenorrhoea from many aspects. NO could regulate uterine contraction and execute pain modulation of peripheral and central levels [38]. Through the NO-CGMP pathway, NO can cause pain and analgesia. The decrease in NO could promote nociceptive transmission and pain, whereas the increase in NO could inhibit analgesia [39]. Calcium ions also play an important role in primary dysmenorrhoea. Intracellular calcium overload induced cell membrane damage and cell energy metabolism; it also contributed to the ischemia-reperfusion injury, causing vasoconstriction of uterine blood vessels (uterine ischemia), increasing uterine smooth muscle contraction, and reducing the endometrial blood supply, which lead to the occurrence of dysmenorrhoea [40].

### 4.3. Open Problems

The following problems need further investigation: whether only the selected pain factors could reflect the results which were consistent with the clinical treatment; whether the association with drug resistance resulted in no difference between the one-cycle group and the last 3 d of cycle group of primary dysmenorrhoea model mice; the main reason and whether it is related to the mechanism of possible hormone regulation. Given the preliminary results of this experimental study, further clinical and animal experimental studies will be conducted.

## 5. Conclusion

Both in one-cycle and three-cycle experiments, comparing with the model control group, the writhing times and contents of PGF_2*α*_ and calcium ion in serum were significantly decreased by administration of JQF (last 3 d group and 6 d group) or Yuanhu Zhitong tablet. Similarly, levels of PGE_2_, NO as well as PGF_2*α*_ and PGE_2_ ratio were normalized in the JQF last 3 d group and 6 d group. Moreover, results in the three-cycle part were the same as that of one cycle, which proved that the cycles and the different times in the same cycle (the last 3 d group and 6 d group) of administration with JQF had the same treatment effect and all remained valid. What we found confirmed the clinical effective treatment of selecting 3 d to 7 d before menstruation instead of the entire physiological cycle therapy in primary dysmenorrhoea treatment. The animal model designed according to the mice oestrous cycle, in this study, may be more feasible and closer to the evaluation of clinical curative effect. It supplies a good foundation for further exploration of in-depth mechanisms of primary dysmenorrhoea treatment.

## Figures and Tables

**Table 1:** Primary dysmenorrhoea mice model and administration.

	Normal	Model	3 d	6 d	Yuanhu
D1	Saline	Oestradiol benzoate	Oestradiol benzoate	Oestradiol benzoate	Oestradiol benzoate
	Saline	Saline	Saline	Jingqian Zhitong Fang (JQF)	Yuanhu Zhitong tablet

D2	Saline	Oestradiol benzoate	Oestradiol benzoate	Oestradiol benzoate	Oestradiol benzoate
	Saline	Saline	Saline	Jingqian Zhitong Fang (JQF)	Yuanhu Zhitong tablet

D3	Saline	Oestradiol benzoate	Oestradiol benzoate	Oestradiol benzoate	Oestradiol benzoate
	Saline	Saline	Saline	Jingqian Zhitong Fang (JQF)	Yuanhu Zhitong tablet

D4	Saline	Oestradiol benzoate	Oestradiol benzoate	Oestradiol benzoate	Oestradiol benzoate
	Saline	Saline	Jingqian Zhitong Fang (JQF)	Jingqian Zhitong Fang (JQF)	Yuanhu Zhitong tablet

D5	Saline	Oestradiol benzoate	Oestradiol benzoate	Oestradiol benzoate	Oestradiol benzoate
	Saline	Saline	Jingqian Zhitong Fang (JQF)	Jingqian Zhitong Fang (JQF)	Yuanhu Zhitong tablet

D6	Saline	Oestradiol benzoate	Oestradiol benzoate	Oestradiol benzoate	Oestradiol benzoate
	Saline	Saline	Jingqian Zhitong Fang (JQF)	Jingqian Zhitong Fang (JQF)	Yuanhu Zhitong tablet

Note: the dosage of each drug was subcutaneous injection of oestradiol benzoate or saline (10 mg/kg); JQF (0.218 g/kg) and Yuanhu Zhitong tablet (0.218 g/kg) were intragastrically administered (0.02 mL/g).

For each group, moulding (saline or oestradiol benzoate) and drugs intervention (saline, JQF, or Yuanhu Zhitong tablet) were almost simultaneously performed in each day.
